# Characteristics and Distribution of Scholarship Donations From Pharmaceutical Companies to Japanese Healthcare Institutions in 2017: A Cross-sectional Analysis

**DOI:** 10.34172/ijhpm.2023.7621

**Published:** 2023-08-21

**Authors:** Anju Murayama, Sae Kamamoto, Hiroaki Saito, Erika Yamashita, Yosuke Suzuki, Tetsuya Tanimoto, Piotr Ozieranski, Akihiko Ozaki

**Affiliations:** ^1^Tohoku University School of Medicine, Sendai, Japan; ^2^Medical Governance Research Institute, Tokyo, Japan; ^3^Department of Internal Medicine, Soma Central Hospital, Soma, Japan; ^4^Department of Internal Medicine, Navitas Clinic Kawasaki, Kawasaki, Japan; ^5^Department of Social and Policy Sciences, University of Bath, Bath, UK; ^6^Department of Breast and Thyroid Surgery, Jyoban Hospital of Tokiwa Foundation, Iwaki, Japan

**Keywords:** Transparency, Japan, Pharmaceutical Payment, Non-research Payment, Institutional Conflicts of Interest, Health Policy

## Abstract

**Background:** Financial relationships between healthcare institutions and pharmaceutical companies can lead to conflicts of interest (COIs), potentially compromising patients’ care. In Japan, scholarship donations, unique type of payments made to healthcare institutions and their subunits by pharmaceutical industries without restricting their use including non-educational or research purpose, may often have implicit promotional purposes. However, detailed information about these payments remains scarce.

**Methods:** This study employed a cross-sectional design to analyse the extent and distribution of all scholarship donations made by all 73 pharmaceutical companies belonging to the Japan Pharmaceutical Manufacturers Association (JPMA) to healthcare institutions in 2017. Data were obtained from publicly available sources from the companies, and the total number of payments, their distributions across various institutions and specialties were analysed.

**Results:** A total of 27 007 payment contracts amounting to $178 703 721 in scholarship donations were made to 4839 specific departments and laboratories at 251 different institutions by 67 pharmaceutical companies. National universities received 50.8% of total payments. All universities setting medical school in Japan received one or more payments. Domestic pharmaceutical companies contributed to $137 797 302 (77.1%) in total. Clinical medicine departments received 89.6% ($160 113 147) with 6.2% ($11 011 946) and 2.0% ($3 600 456) allocated to basic medicine and social medicine specialties, respectively.

**Conclusion:** This study provided a comprehensive overview of scholarship donations from pharmaceutical companies to healthcare institutions in Japan, revealing significant financial support primarily directed to national universities and clinical medicine departments. Japanese policy-makers should consider implementing regulations that promote transparency and mitigate potential COIs arising from scholarship donations, which may be useful in other countries with similar schemes.

## Background

**Key Messages**

**Implications for policy makers**

- There were substantial scholarship donations from pharmaceutical companies to healthcare institutions in 2017, amounting to more than US$ 178 million in Japan.
- Substantial scholarship donations were mostly made to national universities specializing clinical medicine by domestic pharmaceutical companies in Japan.
- Further study should be conducted to indicate whether these scholarship donations help healthcare institutions and professionals to improve research and clinical practice.

**Implications for the public**

Scholarship donation is Japan’s traditional type of payment made to healthcare institutions and their subunits by pharmaceutical industries without restricting their use, and may often have implicit promotional purpose. There were substantial scholarship donations from pharmaceutical companies to healthcare institutions in 2017, amounting to more than UD$ 178 million in Japan. Substantial scholarship donations were mostly made to national universities specializing clinical medicine by domestic pharmaceutical companies in Japan. The debate on quitting scholarship donations without considering this current circumstance might lead to further difficulties in conducting research at Japanese universities. Future studies should assess whether the scholarship donations help healthcare institutions to promote research without playing a role of a bribe from pharmaceutical companies to promote their own products.

Collaborations between pharmaceutical companies and healthcare professionals, healthcare institutions, such as regulatory bodies, hospitals or medical schools, and patient institutions are essential for understanding diseases and the development of novel drugs.^1,2^ However, these collaborations often entail financial relationships leading to conflicts of interest (COIs). In addition to COIs affecting individual healthcare professionals, there are institutional COIs (ICOIs) arising at the level of recipient institutions or their subunits, such as hospital departments.^3^ The National Academy of Medicine in the United States states that “institutional COIs arise when an institution’s own financial interests or those of its senior officials pose risks of undue influence on decisions involving the institution’s primary interests.”^3^ ICOIs have received less attention than individual COIs as they are more difficult to identify and usually conform to legal norms because more stakeholders are involved compared to individual COIs. However, both individual COIs and ICOIs with pharmaceutical companies have repeatedly disturbed patient-centered care, leading to bias in research findings,^4^ influence on drug prescribing patterns^5-7^ and recommendations of clinical guidelines.^8,9^

 One of the most substantial and widespread examples of ICOIs is “scholarship donations” (*Shogaku-kifu* in Japanese) unique to Japan. Scholarship donations are a traditional payment type that, to the best of our knowledge, does not exist outside of Japan.^10^ Although contracts between healthcare institutions and pharmaceutical companies typically undergo formal review at the administrative offices of recipient healthcare institutions, scholarship donations need not document specific purposes, usage, costs, or periods associated with payments. Namely, the term “scholarship donation” may be misleading, as it could imply a direct support for student tuition or training. While these donations may be presented with an educational purpose on the surface, they can also be used for non-educational purposes, such as hiring staff, holding social events, purchasing furniture, etc. Also, it differs from non-research payments for lecturing, consulting, and writing compensations. Unlike “unrestricted educational grants” or “unrestricted research grants,” “scholarship donations” do not necessarily imply a clear educational or research purpose, and their usage may be more flexible and open to potential COIs, underscoring the need to investigate this unique form of industry sponsorship in Japan. Due to these characteristics and flexibility in spending money obtained from pharmaceutical companies, scholarship donations have been vital in supporting the operation of many healthcare institutions in Japan.

 On the other hand, scholarship donations can easily have implicit promotional purposes by pharmaceutical companies, mostly highlighted in adverse cases such as the recent bribery case at the Mie University^10^ and the Diovan scandal.^11^ In the Diovan scandal, Novartis admitted paying about US$ 11 million scholarship donations for funding and honoraria to conduct five clinical trials. However, none of the trials disclosed that they were funded by scholarship donations,^12-17^ nor did they explain to patients the source of funding for the trials.^10^ After this scandal, several pharmaceutical companies, foreign pharmaceutical companies in particular such as Janssen Pharmaceutical K.K., Bristol-Myers Squibb Company, and Celgene Corporation, have quitted or reduced donating scholarship donations. As another example, Astellas Pharma has stopped making scholarship donations and instead has donated to the government agency to allocate their research funds to healthcare institutions and healthcare professionals since 2020. Also, the recent scandal of scholarship donation in exchange for the increased prescription at the Department of Anesthesiology, Mie University has led to discussions about closing the door on scholarship donations in all pharmaceutical companies in Japan, because the scholarship donation was judged as a bribe by the adjudgment in the first instance at a Japanese District Court. Meanwhile, many university hospitals in Japan face difficulties in continuing research due to a decrease in public research funds from the Japanese government.^10^ Under these circumstances, it might be the case that the scholarship donations have been playing a role in supporting healthcare institutions and professionals at universities to continue research in Japan.

 Inappropriately managed COIs between pharmaceutical companies and healthcare sectors have repeatedly triggered medical scandals in Japan.^11^ To manage COIs in line with other developed countries, the Japan Pharmaceutical Manufacturers Association (JPMA) set a transparency guideline in 2011, and based on this guideline, since 2013, full details of scholarship donations and their recipients have been required to be disclosed annually by companies on their websites. We previously reported that the disclosure and collection of payment data from pharmaceutical companies elucidated that a total of $203 380 412 scholarship donations were paid to healthcare institutions from the 71 member companies of the JPMA in 2016, which was as large as the amount of payments to healthcare professionals ($235 958 130) for lecturing, consulting, and writing in 2016.^18^ However, scholarship donations have not yet been analyzed systematically, in contrast to individual-level payments.^11,19-27^

 This study aimed to elucidate the extent and distribution of financial relationships between pharmaceutical companies and healthcare institutions in Japan, using publicly disclosed payment data of scholarship donations from all pharmaceutical companies belonging to the JPMA in 2017.

## Methods

###  Data Collection

 Payment data for the member of the JPMA were published on the website of each company at the time of data collection in 2019. Pharmaceutical companies that adhere to the transparency guideline disclose the amount of their scholarship donations as part of their academic research support expenses. The transparency guidelines are not mandatory, and there is no reporting requirement or penalty for violations when disclosing them, but most companies belonging to the JPMA report in detail. We collected payment data concerning scholarship donations from the 73 companies which belonged to the JPMA in 2017. Using the collected data, we generated a unified single database, as described previously.^19,28^ Our database included the names of hospitals or university departments, the names of pharmaceutical companies, the monetary amounts of the payments, and the frequency of the payments. The unified database was stored by Microsoft Excel, version 16.0 (Microsoft Corp).

###  Data Analysis 

 After structuring the payment database, each payment was categorized, based on additional web searches, by the type of institution (university, research institution, specialty hospital, other hospitals such as community-based or privately-established, or unknown institution), institutional jurisdiction (national, private, or public such as prefectural or municipal), type of pharmaceutical companies (domestic or international), and department specialty.

 First, an individual investigator categorized the payments by the type of institution and institutional jurisdiction by searching the official websites of each institution and department. Second, the two investigators, AM and the investigator independently categorized the payments by specialty referring to the official website of each institution and department. Our original specialty category was based on the Japanese Medical Specialty Board. However, during the specialty classification, to capture the real picture of payments by specialty, the final specialty classification was discussed by AO, HS, and TT, and decided to specialize into 51 main categories including 28 clinical medicines, 12 basic medicines, 8 social medicine, and 3 other subjects related to the hospital sectors. Internal medicine and surgery were further divided into 14 internal medicine subcategories and 7 surgery subcategories.

 As for a department providing both surgical and medical procedures, we categorized it as “general.” For example, a department concerning oncology, which provides both surgical and medical therapy, was categorized as “General Oncology.” For departments containing more than one specialty, multiple classifications were adopted. For departments that we could not find information on official websites or official information, we contacted pharmaceutical companies or affiliated institutions by email for detailed information on the payments and the department. We filled in the department based on the response we received with detailed information from pharmaceutical companies and the institution for all departments. There were cases where we could not identify specialties for multiple payments.

 After the first specialty classification, the two data that AM and an individual investigator were each responsible for were integrated into one dataset. Then the integrated dataset was divided into three parts, and each part was scrutinized by three different reviewers (AM, an individual investigator, and one external research assistant) for the validity of the classification and discrepancies in the classification of departments with the same name. When discrepancies were found, we organized and cleaned the data to meet the final criteria of categorization. Finally, the three separate parts of the data were combined and used for analysis.

 Then we conducted descriptive analysis for the payment data by the type of institution, pharmaceutical companies, and department specialty. Pharmaceutical companies were classified as foreign companies and domestic companies based on the location of the company’s headquarters. A foreign company was defined as a pharmaceutical company whose headquarters was outside of Japan, and a domestic one was vice versa. We calculated the payments at the level of department, institution, pharmaceutical company, and specialty. In the analysis by specialties, when payment was classified in more than one specialty, the payment value was divided according to the number of specialties in which it was classified. Japanese yen was converted into US dollars using the 2017 average monthly exchange rate of ¥112.1 per $1. We conducted all statistical analyses using Microsoft Excel, version 16.0 (Microsoft Corp), and Stata version 15 (Stata Corporation).

## Results

 We identified a total of 27 007 payments concerning scholarship donations worth $178 703 721 (¥20 032 687 112) from 67 pharmaceutical companies (67/73, 91.8%) to 4839 specific departments and laboratories at 251 different institutions in 2017.

Table 1 summarizes the characteristics of 251 institutions receiving scholarship donations and 67 pharmaceutical companies making scholarship donations. A breakdown of the institutions was as follows: 73 private universities (29.1%); 47 national universities (18.7%); 42 national hospitals (16.7%); 16 public universities (6.4%); 11 private research institutes (4.4%); 10 private hospitals (4.0%) and 52 other institutions (20.7%). All 82 medical schools in Japan received one or more scholarship donations (data not shown).

**Table 1:** Characteristics of the Healthcare Institutions and Pharmaceutical Companies Made the Scholarship Donations in 2017

**Variables **	**Institutions Receiving Donations From Japanese Domestic Companies (N = 228 Institutions)**^a^	**Institutions Receiving Donations From Foreign Companies (N=228 Institutions)**^b^	**Overall (N = 251 Institutions)**
Total, No. (%)	228 (90.8)	164 (65.3)	251
University, No. (%)	131 (96.3)	102 (75.0)	136 (54.2)
National	47 (100.0)	42 (89.4)	47 (18.7)
Public	16 (100.0)	11 (68.8)	16 (6.4)
Private	68 (93.2)	49 (67.1)	73 (29.1)
Research institute, No. (%)	24 (96.0)	14 (56.0)	25 (10.0)
National	8 (100.0)	7 (87.5)	8 (3.2)
Public	6 (100.0)	1 (16.7)	6 (2.4)
Private	10 (90.9)	6 (54.5)	11 (4.4)
Special hospital, No. (%)	16 (88.9)	12 (66.7)	18 (7.2)
National	9 (100.0)	5 (55.6)	9 (3.6)
Pubic	5 (71.4)	6 (85.7)	7 (2.8)
Private	2 (100.0)	1 (50.0)	2 (0.8)
Municipal and other hospitals, No. (%)	54 (90.0)	27 (45.0)	60 (23.9)
National	39 (92.9)	21 (50.0)	42 (16.7)
Public	7 (87.5)	4 (50.0)	8 (3.2)
Private	9 (90.0)	2 (20.0)	10 (4.0)
Other institutions	3 (25.0)	9 (75.0)	12 (4.8)
Pharmaceutical companies collecting payment data, N			73 Companies
Domestic			54 Companies
International			19 Companies

^a^ Proportion represents a number of institutions receiving donations from Japanese domestic companies per entire institutions with the same characteristics receiving donations from at least one company. ^b^Proportion represents a number of institutions receiving donations from foreign companies per entire institutions with the same characteristics receiving donations from at least one company.

Table 2 shows the financial characteristics of scholarship donations. At the department level, each department or laboratory received scholarship donations worth of $36 930 on average (standard deviation [SD]: $58 073), and with the median of $16 503 (interquartile ranges [IQR]: $5 352‒$44 603). Similarly, the average and median scholarship donations were $711 967 (SD: $1 283 742) and $32 114 (IQR: $7136‒$1 219 224) per institution, respectively. Also, pharmaceutical companies made payments concerning scholarship donation worth of $2 666 827 (SD: $3 282 833) and $1 151 204 (IQR: $352 783‒$3 061 073) per company in average and median, respectively. 96.5% of payments are concentrated on university departments and laboratories.

**Table 2:** Financial Characteristics of the Scholarship Donation in Japan in 2017

**Variables**	**Payment, $**	**Number of Cases, n**
Total	178 703 721	27 007
Average (SD)		
Department level	36 930 (58 073)	5.6 (6.4)
Institution level	711 967 (1 283 742)	107.6 (158.8)
Pharmaceutical company level^a^	2 666 827 (3 282 833)	385.8 (476.2)
Median (IQR)		
Department level	16 503 (5 352‒44 603)	3 (1‒8)
Institution level	32 114 (7 136‒1 219 224)	5 (1‒231)
Pharmaceutical company level	1 151 204 (352 783‒3 061 073)	213 (39‒558)
Total payments by institution type		
University	172 512 024 (96.5%)	25 974 (96.2%)
National	90 861 713 (50.8%)	12 627 (46.8%)
Public	14 386 619 (8.1%)	2 418 (9.0%)
Private	67 263 692 (37.6%)	10 929 (40.5%)
Research institute	2 169 856 (1.2%)	320 (1.2%)
National	1 085 459 (0.6%)	185 (0.7%)
Public	34 790 (0.0%)	11 (0.0%)
Private	1 049 607 (0.6%)	124 (0.5%)
Special hospital	1 681 980 (0.9%)	263 (1.0%)
National	710 972 (0.4%)	165 (0.6%)
Pubic	792 596 (0.4%)	40 (0.2%)
Private	178 412 (0.1%)	58 (0.2%)
Municipal and other hospitals	1 871 989 (1.1%)	438 (1.6%)
National	1 316 682 (0.7%)	283 (1.0%)
Public	285 459 (0.2%)	84 (0.3%)
Private	269 848 (0.2%)	71 (0.3%)
Other institution	467 868 (0.3%)	12 (0.0%)

Abbreviations: SD, standard deviation; IQR, interquartile range. ^a^Calculations were made among the pharmaceutical companies for which data on scholarship donations was available.

 Data on scholarship donations were collected from 67 of the 73 pharmaceutical companies. This includes 52 domestic companies (96.3% of the 54 total domestic companies) and 15 foreign companies (78.9% of the 19 total foreign companies) (Table 3 and Supplementary file 1). Of the total payments, domestic companies contributed 77.1%, amounting to $137 797 302. In 2017, four foreign companies — Janssen Pharmaceutical K.K., Bristol-Myers Squibb Company, Celgene Corporation, and Shire Japan — did not make payments related to scholarship donations. Similarly, one domestic company, Seikagaku Corporation, did not make such payments. Kyowa Kirin Co., Ltd. made a scholarship donation payment and disclosed it, but we missed collecting the data during its disclosure period. When we later attempted to collect their 2017 payment data, the company started disclosing the 2018 data and declined our request to once again disclose the 2017 data. Chugai Pharmaceutical Co. Ltd. made the largest payment of $13 445 495 to 1325 departments and institutions, followed by Astellas with $11 684 211, Takeda with $10 316 057, Daiichi Sankyo with $9 750 277, and Eisai with $9 365 745. Among the top ten pharmaceutical companies with the largest scholarship donations, eight companies were domestic pharmaceutical companies, while two foreign pharmaceutical companies, MSD and Pfizer made the eighth and ninth largest payments concerning scholarship donations, respectively.

**Table 3:** Scholarship Donation Provided by the Top Five Paying Companies in Japan in 2017

**Top Five Paying Company**	**Payment Amounts (%), $**	**Number of Cases (%), n**
Chugai Pharmaceutical Co., Ltd.	13 445 495 (7.5)	1485 (5.5)
Astellas Pharma Inc.	11 684 211 (6.5)	1455 (5.4)
Takeda Pharmaceutical Company Ltd.	10 316 057 (5.8)	1591 (5.9)
Daiichi Sankyo Company Ltd.	9 750 277 (5.5)	1603 (5.9)
Eisai Co., Ltd.	9 365 745 (5.2)	1590 (5.9)

 As for specialty, 89.6% of scholarship donations concentrated on 28 clinical medicine specialties including internal medicine, surgery, orthopedic surgery, urology, dermatology, and ophthalmology (Supplementary file 2). Among them, internal medicine occupied 46.0% ($82 236 275) of the total scholarship donations from the pharmaceutical companies in 2017, followed by surgery ($18 430 776, 10.3%), orthopedic surgery ($9 055 291, 5.1%), urology ($8 544 900, 4.8%), and dermatology ($6 964 935, 3.9%). Among all 70 specialties including 14 internal medicine and 7 surgical sub-specialties, the amounts made to top-three specialties (endocrinology, cardiology, and gastroenterology) totaled $40 981 299, which was equivalent to 22.9% of the total scholarship donations in 2017. Of all scholarship donations, 6.2% were allocated to 12 basic medicine specialties, including pharmacology, oncology, biochemistry, and medical engineering. Meanwhile, 2.0% were distributed among eight social medicine specialties. (Figure).

**Figure:**
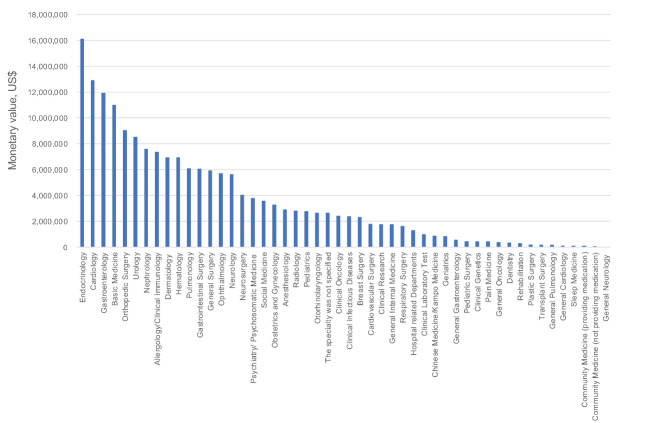
Amounts of the Scholarship Donations Across Major Specialties. While internal medicine and surgeries are presented as distinct subcategories, the 12 basic medicine categories and the 8 social medicine categories were grouped together, respectively. There were cases where we could not identify specialties for multiple payments.

## Discussion

 This study found that a total of $178 703 721 of scholarship donations, which is a major source of non-research ICOIs among Japanese medical society, were paid to 4839 specific departments and laboratories at 251 different institutions by 67, primarily domestic, pharmaceutical companies. The distribution of scholarship donations concentrated on several particular clinical medicine specialties such as endocrinology, cardiology, and gastroenterology. We firstly elucidated Japan’s scholarship donations, although such financial relationships between the pharmaceutical companies and healthcare institutions are different from those of the United States^29,30^ and the United Kingdom.^31-33^ Our previous study found that the scholarship donation accounted for 11.5% of all non-research payments and was equivalent to the payments to healthcare professionals in 2016.^19^ Our current study additionally clarified that a roughly equal amount of scholarship donations were made by pharmaceutical companies in 2016 ($179 937 555) compared to those in 2017 ($178 703 719). Our findings suggest that financial relationships between pharmaceutical companies and healthcare institutions in Japan may continue to persist, suggesting a deeply ingrained custom. It is true that a majority of the pharmaceutical companies are quitting this custom following the recent scandal in Mie University.^10^ However, further longitudinal studies are needed to understand the intentions and motivations of both players in maintaining these relationships.

 First, we found that most of the major pharmaceutical companies in Japan, especially domestic companies, made substantial amounts of scholarship donations to healthcare institutions in 2017. Foreign companies typically conform to Western standards, while Japanese companies often follow traditional home-grown rules. This difference would be particularly relevant in the case of scholarship donations. By distinguishing between foreign and domestic companies, we elucidated the role of these donations in shaping industry payments and the implications for physician-industry relationships in Japan. Despite its unique feature where the scholarship donation does not specify its usage, we can partly analogize it to non-research payments to healthcare institutions in other countries. As for non-research payments to healthcare institutions in the United Kingdom, Ozieranski et al reported that 4028 healthcare institutions received $72 110 157 from 100 pharmaceutical companies in 2015.^31^ The pharmaceutical companies paid $36 487 990 in donations and grants in the United Kingdom. Similarly, Anderson et al found that 1170 US teaching hospitals, equivalent to 91.3% of all teaching hospitals in the United States, received $831 938 468 non-research payments from 529 pharmaceutical companies and medical device manufacturer companies in 2018.^30^ Of all non-research payments made by the companies, 75.2% ($625 700 554) were made for royalty or licenses, and 12.2% ($101 642 765) were for educational purposes. Among the $831 938 468 non-research payments, $315 813 239 (38.0%) were related to biologics or drugs. Therefore, the amount of Japanese scholarship donations in 2017 was 4.90 times higher than the non-research payments from all UK pharmaceutical companies, and 1.76 times higher than the non-research payments from all pharmaceutical companies in the United States, even though the pharmaceutical market size of Japan was less than one-fifth that of the United States (US$ 87.3 billion in Japan vs US$ 508.7 billion in the United States). Given that the US Open Payments Database covers payments from all pharmaceutical and medical device companies and our payment database covered payments from only pharmaceutical companies belonging to the JPMA, the Japanese pharmaceutical companies made much more substantial payments to healthcare institutions as non-research payments than those in other developed countries.

 Second, this study found that $172 512 024 (96.5%) of scholarship donations were distributed to the universities, and all 82 Japanese universities with medical schools received payments from pharmaceutical companies. Considering the nature of medical schools where medical students learn role models for the future, less transparent non-research payments that do not clarify the direct purpose of the payments, such as the scholarship donation, might bias and influence medical education in the universities, clinical practice by university clinicians, and result in less evidence-based treatment leading to wasteful medical spending. In the case of opioid prescription, Anderson et al suggested that the US teaching hospitals should voluntarily prohibit accepting non-research payments from pharmaceutical companies marketing opioids. Indeed, historically, Japan’s scholarship donations have been utilized as hidden research funds and bribes, as in the case of the Diovan scandal^11^ and the Mie University.^10^

 We found that most major domestic pharmaceutical companies paid scholarship donations to healthcare professionals. In this sense, it is also important to recognize that the debate on quitting scholarship donations without considering this current circumstance might lead to further difficulties in conducting research at Japanese universities. We acknowledge that scholarship donations, as described in our study, are a unique custom in Japan and may not be directly applicable to Western countries. However, we believe that understanding this custom and its implications can still provide valuable insights for the international community, especially for non-Western developing countries where similar customs might exist. By sharing the Japanese experience, our study can contribute to a broader understanding of the varied relationships between the pharmaceutical industry and healthcare institutions across different cultural and regulatory contexts including the importance of transparency, industry self-regulation, transparency codes, and regulation of payments from industry to healthcare institutions. Future studies should assess whether scholarship donations help healthcare institutions to promote research without playing a role of a bribe from pharmaceutical companies to promote their own products.

###  Limitations

 This study included several limitations. First, the institution type, nationality of pharmaceutical companies, and specialties of the departments were manually categorized, so human error in the categorizations may be included. Second, since there was no penalty when the pharmaceutical companies did not disclose the payments correctly, the original payment data might have some errors. However, we believe that these issues would have a negligible effect on the overall trends and patterns observed in our study. Finally, the data of non-member companies were not included, but our data sources covered a large majority of the payments made within the industry including most major and influential pharmaceutical companies in the market, which we believe minimizes the potential impact of non-member companies on our findings. Although we cannot provide an exact figure for the number of non-member companies, we estimate that they would account for less than 20% of the market. Given their relatively smaller market share, the influence of these non-member companies on the overall trends observed in our study is likely to be limited.

## Conclusion

 This study unraveled that there were substantial scholarship donations from pharmaceutical companies to healthcare institutions in 2017, amounting to more than US$ 178 million in Japan. Most scholarship donations concentrated on universities with medical school, potentially influencing medical practice, accumulating evidence by conducting research, and medical education in favor of pharmaceutical companies. Further study should be conducted to indicate whether these scholarship donations help healthcare institutions and professionals to improve research and clinical practice.

## Acknowledgements

 This study was funded in part by the Medical Governance Research Institute. This non-profit enterprise receives donations from pharmaceutical companies, including a dispensing pharmacy, namely Ain Pharmacies, Inc., other organizations, and private individuals. This study also received support from Tansa, an independent non-profit news organization dedicated to investigative journalism. The authors also thank Kayo Harada for her dedicated work in obtaining the data. However, none of the entities providing financial support for this study contributed to the design, execution, data analyses, or interpretation of study findings and the drafting of this manuscript.

## Ethical issues

 This study was approved by the Institutional Review Board of the Medical Governance Research Institute. Informed consent was waived from the donated institutions and departments since all the data used in this study were publicly available and did not involve any privacy concerns.

## Competing interests

 HS received personal fees from Taiho Pharmaceutical Co., Ltd. outside the scope of the submitted work. AO receives personal fees from Medical Network Systems, Kyowa Kirin Co., Ltd., and Taiho Pharmaceutical Co., Ltd. outside the scope of the submitted work. TT receives personal fees from Medical Network Systems and Bionics Co. Ltd. outside the scope of the submitted work. The remaining authors declare no conflicts of interest.

## Funding

 This study was funded in part by the Medical Governance Research Institute. This non-profit enterprise receives donations from pharmaceutical companies, including a dispensing pharmacy, namely Ain Pharmacies, Inc., other organizations, and private individuals. This study also received support from Tansa, an independent non-profit news organization dedicated to investigative journalism. However, none of the entities providing financial support for this study contributed to the design, execution, data analyses, or interpretation of study findings and the drafting of this manuscript.

## Supplementary files

Supplementary file 1. Scholarship Donation by Pharmaceutical Companies in Japan in 2017.

Supplementary file 2. Scholarship Donation Distributions Across Specialties in 2017.
