# Supplementary material for: Characteristics and Distribution of Scholarship Donations From Pharmaceutical Companies to Japanese Healthcare Institutions in 2017: A Cross-sectional Analysis
Source: Int J Health Policy Manag. 2023 Aug 21;12:7621. doi: 10.34172/ijhpm.2023.7621 (PMC10590253; doi:10.34172/ijhpm.2023.7621)
Supplement: Supplementary file 1 — Scholarship Donation by Pharmaceutical Companies in Japan in 2017. [file ijhpm-12-7621-s001.pdf]

Article title: Characteristics and Distribution of Scholarship Donations From Pharmaceutical Companies to Japanese Healthcare Institutions in 2017: A Cross-sectional Analysis

Journal name: International Journal of Health Policy and Management (IJHPM)

Authors' information: Anju Murayama[1]*, Sae Kamamoto[2], Hiroaki Saito[3], Erika Yamashita[2], Yosuke Suzuki[2], Tetsuya Tanimoto[4,2], Piotr Ozieranski[5], Akihiko Ozaki[6]

[1]Tohoku University School of Medicine, Sendai, Japan.

[2]Medical Governance Research Institute, Tokyo, Japan.

[3]Department of Internal Medicine, Soma Central Hospital, Soma, Japan.

[4]Department of Internal Medicine, Navitas Clinic Kawasaki, Kawasaki, Japan.

[5]Department of Social and Policy Sciences, University of Bath, Bath, UK.

[6]Department of Breast and Thyroid Surgery, Jyoban Hospital of Tokiwa Foundation, Iwaki, Japan.

***Correspondence to**: Anju Murayama, Email: ange21tera@gmail.com

Citation: Murayama A, Kamamoto S, Saito H, et al. Characteristics and distribution of scholarship donations from pharmaceutical companies to Japanese healthcare institutions in 2017: a cross-sectional analysis. Int J Health Policy Manag. 2023;12:7621. doi:10.34172/ijhpm.2023.7621

Supplementary file 1. Scholarship Donation by Pharmaceutical Companies in Japan in 2017

Ranking	Company name	Total monetary value, $ (%)	Company origin
1	Chugai Pharmaceutical Co., Ltd.	13 445 495 (7.5)	Domestic
2	Astellas Pharma Inc.	11 684 211 (6.5)	Domestic
3	Takeda Pharmaceutical Company Ltd.	10 316 057 (5.8)	Domestic
4	Daiichi Sankyo Company Ltd.	9 750 277 (5.5)	Domestic
5	Eisai Co., Ltd.	9 365 745 (5.2)	Domestic
6	Taiho Pharmaceutical Co., Ltd.	9 235 504 (5.2)	Domestic
7	Ono Pharmaceutical Co., Ltd.	9 125 781 (5.1)	Domestic
8	MSD K.K.	8 645 138 (4.8)	International
9	Pfizer Japan Inc.	7 097 008 (4.0)	International
10	Shionogi & Co., Ltd.	6 004 460 (3.4)	Domestic
11	Mitsubishi Tanabe Pharma Corporation	5 954 505 (3.3)	Domestic
12	Novartis Pharma K.K	5 508 475 (3.1)	International

13	Nippon Boehringer Ingelheim Co., Ltd.	5 013 381 (2.8)	International
14	Otsuka Pharmaceutical Co., Ltd.	4 957 181 (2.8)	Domestic
15	Teijin Pharma Ltd.	3 876 004 (2.2)	Domestic
16	Sumitomo Dainippon Pharma Co., Ltd.	3 743 087 (2.1)	Domestic
17	Eli Lilly Japan K.K.	3 149 866 (1.8)	International
18	Sanofi K.K.	3 089 795 (1.7)	International
19	Asahi Kasei Pharma Corporation	2 974 906 (1.7)	Domestic
20	Terumo Corporation	2 863 515 (1.6)	Domestic
21	Santen Pharmaceutical Co., Ltd.	2 851 918 (1.6)	Domestic
22	Bayer Yakuhin, Ltd.	2 830 071 (1.6)	International
23	Yakult Honsha Co., Ltd.	2 814 451 (1.6)	Domestic
24	AbbVie GK	2 592 756 (1.5)	International
25	Kowa Company, Ltd.	2 561 106 (1.4)	Domestic
26	Torii Pharmaceutical Co., Ltd	2 473 684 (1.4)	Domestic
27	Mochida Pharmaceutical Co., Ltd.	2 051 740 (1.2)	Domestic
28	Kaken Pharmaceutical Co., Ltd.	2 038 359 (1.1)	Domestic
29	Taisho Pharma Co., Ltd.	1 949 153 (1.1)	Domestic
30	Tsumura & CO.	1 917 484 (1.1)	Domestic
31	Nippon Shinyaku Co., Ltd.	1 736 396 (1.0)	Domestic
32	EA Pharma Co.,Ltd.	1 580 731 (0.9)	Domestic
33	Maruho Co., Ltd.	1 514 456 (0.9)	Domestic
34	Novo Nordisk Pharma Ltd.	1 360 393 (0.8)	International
35	Senju Pharmaceutical Co., Ltd.	1 168 599 (0.7)	Domestic
36	Kissei Pharmaceutical Co., Ltd.	1 133 809 (0.6)	Domestic
37	Nippon Kayaku Co., Ltd.	1 127 565 (0.6)	Domestic
38	Kyorin Pharmaceutical Co., Ltd.	1 044 603 (0.6)	Domestic
39	Nihon Pharmaceutical Co., Ltd.	903 015 (0.5)	Domestic
40	Alcon Japan Ltd.	651 204 (0.4)	International
41	Nippon Zoki Pharmaceutical Co., Ltd.	560 214 (0.3)	Domestic
42	Ayumi Pharmaceutical Corporation	553 970 (0.3)	Domestic
43	Merck KGaA	489 295 (0.3)	International
44	Hisamitsu Pharmaceutical Co., Inc.	467 886 (0.3)	Domestic
45	Zeria Pharmaceutical Co., Ltd.	445 138 (0.3)	Domestic
46	ASKA Pharmaceutical Co., Ltd.	440 232 (0.3)	Domestic
47	Pola Pharma Co., Ltd.	433 541 (0.2)	Domestic
48	Meiji Seika Pharma Co., Ltd.	410 348 (0.2)	Domestic
49	Sanwa Kagakuk kenkyusho Co., Ltd.	355 040 (0.2)	Domestic
50	FujiFilm Toyama Chemical Co., Ltd.	354 041 (0.2)	Domestic
51	Kracie Holdings,Ltd.	352 364 (0.2)	Domestic
52	Wakamoto Pharmaceutical Co., Ltd.	347 904 (0.2)	Domestic
53	UCB Japan Co., Ltd.	307 761 (0.2)	International
54	Toray Industries Co., Ltd.	260 285 (0.2)	Domestic
55	Teikoku Seiyaku Co., Ltd.	199 822 (0.1)	Domestic
56	Maruishi Pharmaceutical. Co., Ltd.	133 809 (0.1)	Domestic
57	Baxalta Japan Ltd.	99 911 (0.1)	International
58	Fuso Pharmaceutical Industries, Ltd.	94 558 (0.1)	Domestic

59	Toa Eiyo Ltd.	69 581 (0.0)	Domestic
60	AstraZeneca K.K.	62 444 (0.0)	International
61	Minophagen Pharmaceutical Co., Ltd.	49 063 (0.0)	Domestic
62	Biofermin Pharmaceutical Co., Ltd.	36 574 (0.0)	Domestic
63	Nippon Chemiphar Co., Ltd.	33 006 (0.0)	Domestic
64	Fujimoto Pharmaceutical Corporation	18 733 (0.0)	Domestic
65	Bee Brand Medico Dental. Co., Ltd.	12 935 (0.0)	Domestic
66	GlaxoSmithKline K.K.	8 921 (0.0)	International
67	Kyoto Pharmaceutical Industries, Ltd.	4 460 (0.0)	Domestic
68	Kyowa Kirin Co., Ltd.	0 (0.0)	Domestic
69	Seikagaku Corporation	0 (0.0)	Domestic
70	Janssen Pharmaceutical K.K.	0 (0.0)	International
71	Bristol-Myers Squibb Company	0 (0.0)	International
72	Celgene Corporation	0 (0.0)	International
73	Shire Japan	0 (0.0)	International
