# Supplementary material for: Characteristics and Distribution of Scholarship Donations From Pharmaceutical Companies to Japanese Healthcare Institutions in 2017: A Cross-sectional Analysis
Source: Int J Health Policy Manag. 2023 Aug 21;12:7621. doi: 10.34172/ijhpm.2023.7621 (PMC10590253; doi:10.34172/ijhpm.2023.7621)
Supplement: Supplementary file 2 — Scholarship Donation Distributions Across Specialties in 2017. [file ijhpm-12-7621-s002.pdf]

Article title: Characteristics and Distribution of Scholarship Donations From Pharmaceutical Companies to Japanese Healthcare Institutions in 2017: A Cross-sectional Analysis

Journal name: International Journal of Health Policy and Management (IJHPM)

Authors' information: Anju Murayama[1]*, Sae Kamamoto[2], Hiroaki Saito[3], Erika Yamashita[2], Yosuke Suzuki[2], Tetsuya Tanimoto[4,2], Piotr Ozieranski[5], Akihiko Ozaki[6]

[1]Tohoku University School of Medicine, Sendai, Japan.

[2]Medical Governance Research Institute, Tokyo, Japan.

[3]Department of Internal Medicine, Soma Central Hospital, Soma, Japan.

[4]Department of Internal Medicine, Navitas Clinic Kawasaki, Kawasaki, Japan.

[5]Department of Social and Policy Sciences, University of Bath, Bath, UK.

[6]Department of Breast and Thyroid Surgery, Jyoban Hospital of Tokiwa Foundation, Iwaki, Japan.

***Correspondence to**: Anju Murayama, Email: ange21tera@gmail.com

Citation: Murayama A, Kamamoto S, Saito H, et al. Characteristics and distribution of scholarship donations from pharmaceutical companies to Japanese healthcare institutions in 2017: a cross-sectional analysis. Int J Health Policy Manag. 2023;12:7621. doi:10.34172/ijhpm.2023.7621

Supplementary file 2. Scholarship Donation Distributions Across Specialties in 2017

Variables	Total monetary value, $ (%)
Clinical medicine	160 113 147 (89.6)
Internal medicine	82 236 275 (46.0)
Endocrinology/Diabetology	16 125 216 (9.0)
Cardiology	12 930 607 (7.2)
Gastroenterology	11 925 477 (6.7)
Nephrology	7 612 623 (4.3)
Allergology/Clinical immunology	7 370 109 (4.1)
Hematology	6 941 469 (3.9)
Pulmonology	6 101 024 (3.4)
Neurology	5 646 820 (3.2)
Clinical oncology	2 428 784 (1.4)
Infectious diseases	2 401 398 (1.3)
General internal medicine	1 768 213 (1.0)
Geriatrics	855 635 (0.5)
Community Medicine (providing medical treatment)	102 141 (0.1)
Breast medicine	26 762 (0.0)
Surgery	18 430 776 (10.3)
Gastrointestinal surgery	6 078 791 (3.4)
General Surgery	5 939 622 (3.3)
Breast surgery	2 340 953 (1.3)
Cardiovascular surgery	1 795 904 (1.0)
Respiratory surgery	1 636 493 (0.9)
Pediatric surgery	460 155 (0.3)

Transplant surgery	178 858 (0.1)
Orthopedic surgery	9 055 291 (5.1)
Urology	8 544 900 (4.8)
Dermatology	6 964 935 (3.9)
Ophthalmology	5 704 936 (3.2)
Neurosurgery	4 046 833 (2.3)
Psychiatry/ Psychosomatic medicine	3 797 056 (2.1)
Obstetrics and gynecology	3 293 116 (1.8)
Anesthesiology	2 921 687 (1.6)
Radiology	2 822 777 (1.6)
Pediatrics	2 783 848 (1.6)
Otorhinolaryngology	2 671 537 (1.5)
Clinical research	1 781 891 (1.0)
Clinical laboratory test	1 012 935 (0.6)
Chinese medicine	879 572 (0.5)
General gastroenterology	578 055 (0.3)
Clinical genetics	435 772 (0.2)
Pain medicine	433 541 (0.2)
General Oncology	386 262 (0.2)
Dentistry	364 275 (0.2)
Rehabilitation	307 761 (0.2)
Plastic Surgery	203 539 (0.1)
General pulmonology	173 506 (0.1)
General cardiology	110 616 (0.1)
Sleep medicine	105 263 (0.1)
General community medicine	54 416 (0.0)
General neurology	11 775 (0.0)
Basic medicine	11 011 946 (6.2)
Pharmacology	2 148 677 (1.2)
Oncology	1 868 421 (1.0)
Biochemistry	1 681 088 (0.9)
Medical engineering	1 258 311 (0.7)
Immunology	1 136 099 (0.6)
Regenerative medicine	850 580 (0.5)
Physiology	836 753 (0.5)
Microbiology	503 650 (0.3)
Nutritional science	339 429 (0.2)
Pathology	322 480 (0.2)
Neuroscience	44 603 (0.0)
Anatomy	21 855 (0.0)
Social medicine	3 600 456 (2.0)
Public health	1 820 250 (1.0)
Medical education	724 353 (0.4)
Preventive medicine	455 943 (0.3)
Health science	308 653 (0.2)
Medical safety	135 593 (0.1)
Health center	104 371 (0.1)
Medical ethics	44 157 (0.0)
Forensic medicine	7 136 (0.0)
Hospital related departments	1 309 438 (0.7)
Hospital pharmacy	937 556 (0.5)
Hospital nurse	284 567 (0.2)
Hospital office	87 315 (0.0)
The specialty was not specified	2 668 733 (1.5)
